# Beyond Bushfires: Community, Resilience and Recovery - a longitudinal mixed method study of the medium to long term impacts of bushfires on mental health and social connectedness

**DOI:** 10.1186/1471-2458-13-1036

**Published:** 2013-11-04

**Authors:** Lisa Gibbs, Elizabeth Waters, Richard A Bryant, Philippa Pattison, Dean Lusher, Louise Harms, John Richardson, Colin MacDougall, Karen Block, Elyse Snowdon, Hugh Colin Gallagher, Vikki Sinnott, Greg Ireton, David Forbes

**Affiliations:** 1 Jack Brockhoff Child Health and Wellbeing Program, McCaughey VicHealth Centre for Community Wellbeing, University of Melbourne, Level 5, 207 Bouverie Street, Carlton, VIC 3053, Australia; 2 School of Psychology, University of New South Wales, Sydney, NSW 2052, Australia; 3 Melbourne School of Psychological Sciences, University of Melbourne, Melbourne, VIC 3052, Australia; 4 Swinburne Institute for Social Research, Faculty of Life and Social Sciences, Swinburne University of Technology, 400 Burwood Road, Hawthorn, VIC 3122, Australia; 5 Department of Social Work, University of Melbourne, L6 Alan Gilbert Building, Carlton, VIC 3053, Australia; 6 Emergency Services, Australian Red Cross, 155 Pelham Street, Carlton, VIC 3053, Australia; 7 Southgate Institute for Health, Society and Equity, and School of Medicine, Flinders University, GPO Box 2100, Adelaide, South Australia 5001, Australia; 8 Prevention and Population Health Branch, Department of Health, Melbourne, VIC 3000, Australia; 9 Health and Human Services Emergency Management, Department of Human Services, Melbourne, Vic 3000, Australia; 10 Australian Centre for Posttraumatic Mental Health, University of Melbourne, East Melbourne, VIC 3002, Australia

**Keywords:** Disasters, Social networks, Mental health, Epidemiologic methods, Qualitative research, Community-based participatory research

## Abstract

**Background:**

Natural disasters represent an increasing threat both in terms of incidence and severity as a result of climate change. Although much is known about individual responses to disasters, much less is known about the social and contextual response and how this interacts with individual trajectories in terms of mental health, wellbeing and social connectedness. The 2009 bushfires in Victoria, Australia caused much loss of life, property destruction, and community disturbance. In order to progress future preparedness, response and recovery, it is crucial to measure and understand the impact of disasters at both individual and community levels.

**Methods/design:**

This study aims to profile the range of mental health, wellbeing and social impacts of the Victorian 2009 bushfires over time using multiple methodologies and involving multiple community partners. A diversity of communities including bushfire affected and unaffected will be involved in the study and will include current and former residents (at the time of the Feb 2009 fires). Participants will be surveyed in 2012, 2014 and, funding permitting, in 2016 to map the predictors and outcomes of mental health, wellbeing and social functioning. Ongoing community visits, as well as interviews and focus group discussions in 2013 and 2014, will provide both contextual information and evidence of changing individual and community experiences in the medium to long term post disaster. The study will include adults, adolescents and children over the age of 5.

**Discussion:**

Conducting the study over five years and focussing on the role of social networks will provide new insights into the interplay between individual and community factors and their influence on recovery from natural disaster over time. The study findings will thereby expand understanding of long term disaster recovery needs for individuals and communities.

## Background

Changing patterns in global climate have resulted in growing awareness that natural disasters will probably increase in the coming years, resulting in more bushfires, severe storms, and flooding that can pose a threat to individual and social well-being [1]. For example, bushfires and other hazards are expected to present an increased threat to Australian communities over the coming years, with more days with a high risk of fire expected as a result of climate change [2,3]. The catastrophic bushfires of February 2009, including the 'Black Saturday’ fires of February 7, led to tragic loss of life and far-reaching damage to the landscape and physical infrastructure. One hundred and nine communities self-identified as being impacted by bushfires. There were 173 fatalities, 3,500 buildings (2,133 houses) damaged or destroyed, and a significant assault on the social infrastructure within communities [4]. In order to progress future preparedness, response and recovery, it is crucial to measure and understand the impact of disasters at both individual and community levels.

There is converging evidence that massive disasters contribute to impaired mental health and social disruption [5,6]. However, our current knowledge base is very limited by several factors that impede optimal preparation to respond to the health and wellbeing consequences of a disaster. First, there is an urgent need to examine further the inter-related factors that generate recovery following disaster. To date, research has focused either on the mental health functioning of individuals [7]*or* economic impacts. This approach has been limiting because disasters, by definition, affect individuals within a broader social context, and therefore full understanding of the nature of disaster response requires that both individual and community factors must be studied in concert. Second, the majority of disaster research has focused on the relatively short-term effects of disaster [7-9]. This is a major limitation because impacts may be experienced over long periods of time as social networks respond to, are re-established and evolve following a disaster. For example, evidence following Hurricane Katrina demonstrated that mental health functioning deteriorated markedly at longer-term follow-up assessments, arguably as social and infrastructure support was not available [10]. Third, most disaster research has attended to adult survivors, with a relative neglect of children and adolescents, let alone combining a focus on adults, children and young people in the same study. This is a critical issue because indirect evidence suggests that distressing events may impact on developmental progress in younger people, which can only be properly studied through longitudinal study over the course of years [11,12]. Fourth, most disaster research focuses solely on specific pathogenic impacts of trauma, and their associated risk factors. Accordingly, risk is typically treated as an additive combination of sociodemographic variables [9] rather than as systems of ongoing social arrangements which might be amenable to population-level intervention. While such combinations of risk factors may help to predict with whom pathology is likely occur, they do not explain how the pathology arose in the first place, or how its presence/absence contributes to a larger picture of an individual’s wellbeing. Ultimately, the critical link between trauma and resilience is left under-examined.

This paper presents the protocol for the 'Beyond Bushfires: Community, Resilience and Recovery’ study (referred to originally in funding documents as Bushfires, Mental Health and Social Connectedness). In the wake of the Victorian bushfires in 2009, this study brings together academic, community, health, government and emergency response organisations to address each of the aforementioned limitations in the evidence. The study will consist of longitudinal, mixed method analyses of social connectedness, mental health functioning and wellbeing in affected and unaffected areas in people of all ages. Importantly, it will identify the major community, family, and individual factors that will enhance adaptation following future disasters. The complexity of the term 'community’ is recognised [13]. It is used in this paper to refer to predominantly geographically bound localities but also includes those residents who have relocated since the February 2009 bushfires [14]. A multidisciplinary approach will account for the interacting social determinants of health and wellbeing post-disaster. The development of the protocol has thus required consideration of study methods that bridge paradigms and scientific disciplines and requires that the varying nature of the data generated are able to be cross referenced and “speak to each other” allowing for more integrated findings. A partnership approach with government and agency partners ensures inclusion of policy and service delivery expertise. An ethnographic approach involving ongoing community liaison will also allow for inclusion of community expertise and contextual information in the development of study design, methods and measures, and interpretation of findings. These multi-sectoral contributions will continue throughout the study.

### Aims

The goal of this study is to support the development of evidence-based strategies for promoting mental health, wellbeing and social inclusion of individuals and communities in regions affected by disasters. The study aims to achieve this by identifying patterns of individual and community recovery from the recent bushfire disaster in Victoria, and factors that enhance recovery. Over a period of 3–8 years post-bushfire, we will work closely in partnership with government, industry and community partners to:

• Describe individual recovery trajectories in terms of mental health, wellbeing and social connectedness

• Increase understanding of individual social needs and the role of social networks in supporting resilience at individual and community levels

• Increase understanding of the particular needs of children and adolescents

• Identify individual and community predictors of recovery from disaster.

### Theoretical framework

This study will be conducted within a socio-ecological theoretical framework which recognises the proximal and distal influences on individual health and health behaviours, as well as the interdependence between individual cognitive and behavioural processes and societal factors on psychological and health outcomes [15]. It will therefore draw on both cognitive and behavioural models, as well as theories of social capital and social network analysis to characterise these interdependencies in terms of relationships within and across groups in community settings [16-21]. A community based participatory approach will be employed that involves the partner organisations, community members and local government as active partners throughout the research process to ensure the relevance and sensitivity of the study as well as translation of study findings into policy, service and community outcomes [22,23]. This approach directly accords with the National Principles for Recovery, and recognition following the Victorian fires of the priority that needs to be given to community engagement in the recovery and reconstruction following a disaster [24].

## Methods/Design

A mixed method, longitudinal study will be conducted to increase understanding of the impact of community members’ bushfire experiences, their recovery needs and how individual and community adaptation shifts over time. The methods include an ethnographic approach, epidemiological and social network surveys, in-depth qualitative interviews and focus group discussions. The study aims to achieve its goals through progressive individual and community measurements over a five-year period in the first instance. Our expectation is that we will continue to collaborate with these individuals and communities beyond the funded five-year period of the study.

### Study location

Selection of Victorian rural communities to be invited to participate in the study was determined according to a purposive sampling model to capture a diversity of experiences as represented by a range of variables. This included level of impact from the February 2009 fires (see Table 1), and demographics as indicated by the SEIFA Index of Relative Socio-Economic Advantage and Disadvantage (IRSAD) from the Australian Bureau of Statistics 2006 Census.^a^ Community selection variables also included population size, region and distance from Melbourne. Community visits were conducted to determine if there was local support for the study to sustain a partnership approach and to check the proposed boundaries for local relevance. As a result, 26 communities in 10 locations in Victoria were identified as participating communities (see Table 2).

**Table 1:** **Fire impact**^**1**^

**Fire impact**	**Features**
High	Fatalities and many houses lost
Medium	Upper end of range may include small number of fatalities through to communities with no fatalities but significant amount of property damage
Low	No fatalities, minimal or no property loss

^1^This is a modified version of the classification system developed for the CFA post-fire analysis of the efficacy of the Community Fireguard Program (Gibbs et al., *CFA Post-fire Qualitative Research: Preliminary Report*. The McCaughey Centre: VicHealth, 2009 p. 6, http://cfa.vic.gov.au/fm_files/attachments/Publications/Post_Fire_Qualitative_Research_Preliminary_Report.pdf).

**Table 2:** Participating communities

**LGA**	**PCP**	**Towns**	**Pop 2006**	**Households**	**SEIFA 2006**	**Impact**	**Age 4–10 years**	**Age 11–17 years**	**Age 18+ 2006**
**PILOT:**									
Greater Bendigo	2	West Bendigo	362	144	5	M	42	43	277
Baw Baw	4	Darnum	561	221	6	L	73	63	425
**MAIN STUDY:**									
Murrindindi, Nillumbik & Whittlesea	3 & 5	Kinglake, Kinglake Central, Kinglake Pheasant Creek	3048	1042	7	H	414	375	2259
	3	Marysville	429	207	4	H	37	36	356
		Buxton	377	142	5		25	51	301
Murrindindi		Taggerty	581	233	4		56	25	500
		Narbethong	271	103	4		22	25	224
		Granton	n/a	n/a	n/a		n/a	n/a	n/a
Yarra Ranges	1	Steels Creek	n/a	n/a	8	M/H	n/a	n/a	n/a
		Dixons Creek	575	222	8	M	54	45	476
Alpine	6	Mudgegonga, Dederang & Glen Creek Kancoona Running Creek, Gundowring & Rosewhite	984	360	2 & 8	M	98	107	779
Indigo		Bruarong							
Latrobe	4	Callignee	485	164	5	H	49	68	368
		Koornalla	n/a	n/a	5	H	n/a	n/a	n/a
Greater Bendigo	2	Axedale	225	89	8	L	21	36	168
Murrindindi	3	Eildon	1148	438	2	L	83	91	974
Mitchell	3	Tallarook	746	297	5	L	65	95	586
**Total Populations:**			**9,792**	**3,662**			**1,039**	**1,060**	**7,693**

Population data is based on the 2006 Census data. Http://www.abs.gov.au/websitedbs/censushome.nsf/home/communityprofiles?opendocument&navpos=230.

Impact ratings as described in Table 1.

PCPs that are partners in study (relevant LGAs in bold).

1. Outer East Health and Community Support Alliance - municipalities of Knox, Maroondah and **Yarra Ranges**.

2. Bendigo Loddon Primary Care Partnership - **City of Greater Bendigo** and the Loddon Shire.

3. Lower Hume Primary Care Partnership – **Mitchell** and **Murrindindi** Shires.

4. Central West Gippsland Primary Care Partnership - municipalities of **Baw Baw** and **Latrobe**.

5. North East Primary Care Partnership (Previously: Banyule Nillumbik Primary Care Alliance) – **Nillumbik**, Banyule and Darebin.

6. Central Hume Primary Care Partnership - **Alpine** and Mansfield Shires and the Rural Cities of Benalla and Wangaratta.

### Sample and recruitment

The study aims for saturation sampling within participating communities to meet methodological requirements of both the epidemiological and social network components of the study. All adults, adolescents and children currently living in the selected communities or who were living in those communities at the time of the 2009 fires, will be eligible and invited to participate in the study. The 2006 census data indicated a total population of 9,792 in the selected communities, including adults, adolescents and children aged 4 and above. The sample for this study will be obtained using contact details gathered by the Victorian Electoral Commission in order to be able to identify the contact details of both current residents and those who have relocated - approximately 7,500 adults in total. A personalised letter of invitation will be sent to the adults in this sample. Children between the ages of 5 and 18 will also be eligible for participation in the study, subject to parent approval. Additional community awareness, news media and social media activities will also be conducted to ensure eligible participants are aware of the opportunity to participate in the study.

### Consent

Parents/caretakers will provide consent for children and will complete the survey on their behalf. Children (primary school students – approx. 5–12 years) who are invited to participate in interviews or focus group discussion will be asked to provide verbal consent in addition to their parent’s written consent. Adolescents (secondary school students, approx. 13–18 years) will be sent a letter of invitation to participate, subject to parent/caretaker consent, and will then provide their own consent.

### Data collection

The various methods being employed, that is, an ethnographic approach, epidemiological and social network surveys, in-depth qualitative interviews and focus group discussions, will require different data collection processes, as detailed below. The qualitative components of the study will adhere to the **RATS guidelines** on qualitative research (http://www.biomedcentral.com/ifora/rats).

### Community visits

In keeping with the ethnographic approach of the study, ongoing community visits were conducted in the developmental stages of the study and will continue throughout. Initially, these were conducted to establish community contacts as a means of information exchange, recognising community groups and members as the source of valued on-site community expertise and a connection with local information networks to support study information dissemination. The input received on the community visits helped in establishing the study name, terminology and branding, the boundaries of the selected communities, the recruitment and data collection methods, and the survey content. The stories being shared by community members with the researchers also provide critical contextual information which will inform the qualitative components of the study.

### Data collection services

A company has been employed to conduct quantitative data collection using computer assisted telephone and web based surveys on behalf of the University. This arrangement is subject to strict privacy and confidentiality guidelines.

### Survey

The survey instrument will be administered in 2012, 2014 and (subject to funding) in 2016 to enable measurement of change over time. The survey has been developed across key themes:

• Mental health and related behaviour

• Social networks

• Relationships with organisations

• Community attitudes

• Physical health

• Wellbeing

• Demographic information

• Bushfire exposure and impact.

Where possible, existing scales were used within the survey, however due to the unique aspects of the study, in some cases it was necessary to develop new questions for inclusion (see Table 3).

**Table 3:** Sources of survey items

**Survey item topic ❖**	**Source**	**Specific reference**
Demographics	ABS Census 2006-2008	
Social networks	Proposed by Pattison, Robins, Y. Kashima, Lusher, E. Kashima, Daraganova & Quintane.	
Fire exposure and impacts	Developed for this study	
General health	Standard self assessment scale	
PTSD	PTSD checklist	Bliese, P. D., Wright, K. M., Adler, A. B., Cabrera, O., Castro, C. A., & Hoge, C. W. (2008). Validating the primary care posttraumatic stress disorder screen and the posttraumatic stress disorder checklist with soldiers returning from combat. Journal of Consulting and Clinical Psychology, 76(2), 272–281.
Depression	PHQ-9	Kroenke K, Spitzer RL, Williams JB. The PHQ-9: Validity of a brief depression severity measure. J Gen Intern Med. 2001;16:606–613.
Psychiatric disorder/psychological distress	K-6	Kessler, R. C., Barker, P. R., Colpe, L. J., Epstein, J. F., Gfroerer, J. C., Hiripi, E., Howes, M. J., Normand, S.-L. T., Manderscheid, R. W., Walters, E. E., & Zaslavsky, A. M. (2003). Screening for serious mental illness in the general population. Archives of General Psychiatry, 60:184–189.
Anger	Modified version of questions on the Anger Attacks Questionnaire	Winkler D, Pjrek E, Kindler J, Heiden A, Kasper S. Validation of a Simplified Definition of Anger Attacks. Psychotherapy and Psychosomatics. 2006;75:103–6.
Alcohol abuse	AUDIT-C	Bush, K., Kivlahan, D., McDonell, M., Fihn, S., & Bradley, K. (1998). The AUDIT Alcohol Consumption Questions (AUDIT-C): An Effective Brief Screening Test for Problem Drinking. Archives of Internal Medicine, 158, 1789-1795
Grief	Prolonged Grief Inventory	Adapted from Shear, Katherine M.; McLaughlin, Katie A.; Ghesquiere, Angela; Gruber, Michael J.; Sampson, Nancy A.; Kessler, Ronald C. *Depression & Anxiety (1091–4269).* Aug2011, Vol. 28 Issue 8, p648-657. 10p. doi:10.1002/da.20865.
Resilience	Connor-Davidson Resilience Scale	Connor, K. & Davidson, J. (2003)
Attachment	Derived from the Experiences in Close Relationships Scale	Olssøn I, Sørebø Ø, Dahl AA. The Norwegian version of the Experiences in Close Relationships measure of adult attachment: Psychometric properties and normative data. Nord J Psychiatry. 2010;64(5)
Wellbeing	Derived from The Australian Unity Wellbeing Index - Community Indicators Victoria (CIV)	Cummins R, Eckersley R, Pallant J, Vugt JV, Misajon R. Developing a National Index of subjective Wellbeing: The Australian Unity Wellbeing Index Social Indicators Research. 2003;64:159-90
Community hope	Proposed by Pattison, Robins, Y. Kashima, Lusher, E. Kashima, Daraganova & Quintane.	
Community cohesion	Questions derived from the Neighbourhood cohesion scale	Buckner J. The development of an instrument to measure neighborhood cohesion. American Journal of Community Psychology. 1988;16(6):771-91

**❖**A copy of the 2012 survey items can be accessed at http://www.beyondbushfires.org.au.

Adapted versions of the survey were developed for adolescents (defined for the purposes of the study as those of secondary school age) for self completion, and for children (defined as primary (elementary) school age) – for parent/guardian completion. Preschool children are not eligible for the study as they would have been babies or not born yet when the fires occurred. The adult and child surveys were piloted first to ensure comprehensibility and acceptability as part of the pilot phase involving two of the selected communities. As no adolescents were recruited in the pilot phase, the adolescent survey was only able to be piloted for comprehensibility with adolescents known to the researchers and not from bushfire affected areas.

Survey data will be collected via an online survey or a telephone interview that will take approximately forty minutes for completion. The mode of survey administration that is, by telephone interview or online, will be determined by participant choice.

### Potential for distress

In recognition of the potential for distress triggered by participation in the study, information will be provided to all participants about support services available to them, including 24 hour options. A suicide protocol and an abuse protocol have been developed in consultation with the Australian Centre for Posttraumatic Mental Health in the unlikely event that risk of either emerges during the course of data collection.

### Interviews and family focus groups

A sub-sample of adults, adolescents and children will be invited to participate in in-depth individual or group interviews in 2013. This component is being included to increase understanding of recovery experiences from the perspective of the participants and to identify any recovery issues not being captured by the structured measures. Purposive sampling will be used initially to seek a diversity of experiences. As themes emerge from the analysis further sampling may occur to allow confirmation of findings or to clarify disconfirming evidence. These interviews will include a walking tour of the local area with the participant/s leading the researcher, and use of a photo description and ordering approach to explore and identify significant local places and activities, and to build an understanding of attachment to community generally. For those who are comfortable engaging in bushfire related discussions, we will also discuss how this attachment to local spaces has been affected by the impact of bushfires and recovery activities. This methodology has been specifically developed to be suitable for children and young people [25]. Walking tours and photo-elicitation techniques have been found to be effective and non-threatening in discussions with children, refugees and participants with limited language skills [26-28]. These methods have also been successfully trialled in developing countries to index children’s perceptions of a traumatic event and its aftermath and to involve them in disaster risk reduction planning [29]. The existing connections with community groups will also provide an opportunity to discuss and build on the understandings developed, to identify other groups or individuals who could make a contribution to the study, and to monitor shifts in the issues being experienced in communities as time passes.

### Analyses

The study will integrate the strengths of two quantitative research paradigms with qualitative thematic analyses:

(a) Structural Equation Modelling (SEM) for prediction of mental health recovery across time;

(b) Cross-sectional and dynamic social network models for the interdependence and co-evolution of social relationships and individual and community outcomes;

(c) Inductive thematic analyses of community visits, in-depth interviews and focus group discussions.

All of the study partners will be involved in the interpretation of the findings to ensure that different perspectives are considered and understood.

(a) Structural equation modelling (SEM) Apart from calculating prevalence rates of primary problems, a key analysis of interest will involve SEM that will treat indices of mental health and functioning as outcome measures and the range of social, family, and individual factors as predictors. The essential outcomes from the initial phase of the study will emerge from SEM analyses that will identify the key risk factors, triggering events, and mediating variables that lead to adaptation in the communities and contribute to sound mental health. The power of the SEM analytic approach with this sample is that it can *specifically* identify the direct and indirect pathways to difficulties and resilience in the affected communities. For example, it may be that a specific risk factor for poor mental health outcomes is counteracted by other protective factors; SEM will allow us to identify the relative contributions of these different factors. This is the essential requisite step that will allow evidence-informed programs to promote recovery in these communities.

(b) Social Network Analysis (SNA) The second significant research intention is concerned with quantitative measurement of the social relations among community members and the impact of social connectedness on individual mental health and overall community outcomes. This form of analysis will be used in those communities where adequate response rates are achieved. While for most network studies, at or near saturation is desirable for the social network analysis, participation rates that are significantly lower than saturation can be accommodated through other approaches, such as conditional estimation approaches that can be implemented on only a sample of the network, or methods for estimating missing network data as well as model parameters. An important element of the measurement approach is that we not only ask *whether* someone receives or gives social support, but also *from whom* they receive help and *to whom* they give it. In order to distinguish between both positive and negative social support, we will also ask participants to identify any person/s or organisation/s that make them feel upset, or make it difficult to receive practical assistance. The measurement approach is done in a way that affords matching of named individuals across respondents (while also respecting individuals’ privacy). As a result, we can obtain a profile of relationships underpinning the giving and receiving of support. We propose to examine social connectedness using statistical network models, which accommodate both cross-sectional and longitudinal network data (specifically, *exponential random graph models* and *network co-evolution models*[30]). Going beyond the very simple technique of counting social ties or asking about general perceptions of social support, these models provide detail regarding mechanisms of social selection and influence that affect the observed patterns of social relationships within an affected community. This approach thus allows to us to examine whether individual mental health and other characteristics are linked to specific network substructures in which individuals are embedded.In this way we can expand our understanding of mental health deficits as affecting not only the individual and his/her family, but also as potentially linked to the wider pattern of social connectedness within a community.

(c) In-depth interview and thematic analysis An inductive, thematic analysis of the in-depth interviews will be conducted by coding and categorising the issues as they arise, conducting within and across category comparisons to identify patterns and themes, and sampling further to allow clarification of emerging or contradictory evidence and to confirm findings. This descriptive analysis will be considered and developed further in reference to the themes informing the survey instrument and identified in the existing evidence base. The subsequent conceptual analysis will then be considered in relation to existing social and health theoretical frameworks to develop a theoretical understanding of the needs and experiences of the study participants that can be applied more generally to bushfire survivors. This will illuminate the survey findings and provide a greater understanding of factors likely to enhance resilience for individuals and communities following bushfires and other disasters.

### Ongoing bushfire activity

It is recognised that threatened or actual bushfire activity or other significant event may occur in the participating communities at some stage throughout the study. The study will continue as a means of capturing the impact of these events, utilising the strong community partnerships to ensure the study progresses sensitively and is adaptable where required to community needs.

### Mixed method analyses

The quantitative and qualitative components will be conducted sequentially, with baseline quantitative data informing sampling for the initial qualitative interviews. Each component will then be analysed independently, with merging of data occurring at the final interpretation stage to extend the breadth and depth of understandings [31].

### Data access

Due to the extremely high value of this data, a copy of the survey data will be kept indefinitely at the Australian Data Archive in Canberra (http://www.ada.edu.au) which stores many important study collections for future research. There will be strict controls over who can see that data and what it can be used for.

### Study outcome and outputs

The **Outcome** of this study will be the development of new knowledge about community based strategies likely to maximise long term community and individual recovery from bushfire disaster.

The study **Outputs** will include:

• A range of methodological and outcomes research papers to contribute to the international disaster evidence base

• A review of policy and service delivery by study partners, informed by the study findings

• A list of recommendations about specific community-based strategies to enhance community and individual recovery post-bushfire disaster

• Ongoing multisector partnerships to support further research, trials and evaluations.

### Ethics approval

Ethics approval for this study was granted by the University of Melbourne Human Research Ethics Committee and the Victorian Department of Education and Early Childhood Development Research Committee.

## Discussion

Climate change is expected to lead to increased natural disasters in the years ahead [3]. For this reason a greater knowledge-base is urgently needed to shape policy for disaster preparedness and response.

In terms of innovation, this study extends many previous epidemiological studies that have addressed prevalence rates of established disorders by:

• Profiling the broad range of problems that can develop following disaster;

• Closely characterising the dynamic relationship between community functioning and mental health outcomes;

• Tracking the impact of the disaster, and the mobilisation of social resources, and the implications for children and adolescents;

• Establishing a platform for long-term surveillance that will track mental health, wellbeing and social trajectories over the medium to long term post-disaster.

These advances are highly innovative because they set the understanding of both community and individual level factors within a systemic context rather than adopting the traditional medical model that narrows attention to individual functioning. Apart from being highly relevant to communities that were largely destroyed or disrupted by the fires in Victoria, this approach will markedly extend current models of disaster response by the development of a coherent account of both community and individual response factors. This represents an important development internationally in the conceptualisation of emergency management because it offers a systematic empirical base for the first time. Recent consensus meetings following massive disasters in which the Chief Investigators have been involved (e.g. the 2004 Asian tsunami, Hurricane Katrina) have pointed to the critical need for studies that provide longitudinal evidence of factors that will lead to better community and individual outcomes following disaster. At a more local level, this study represents an extremely significant advance in identifying the needs of those directly affected by the Victorian bushfires, and enabling evidence-based planning and service delivery by the partner organisations.

Specifically, this study incorporates a combination of a social network analysis, an assessment of community attachment, and an epidemiological study that will allow detailed characterisations of the interplay of community, interpersonal and individual factors in shaping community and individual outcomes. The way in which people form close personal relationships, often referred to as 'attachment’, is receiving interest as an important factor in recovery from disaster and trauma [32]. An 'Experiences in Close Relationships Scale’ has been chosen to assess this construct and analysis. This provides an ideal link between social networking and mental health analysis. These analyses will lead to characterisations that go beyond the simple explanation of social relationships being important for recovery. In particular, using new statistical methods for dynamic, interdependent social processes, we will develop both descriptive and predictive accounts of the impact on community and individual well-being of a variety of individual factors, as well as pre-existing and emerging aspects of social connectedness. This approach will assist us to establish a dynamic map of the pathways that generate, exacerbate, moderate and protect against adverse outcomes in people who experience a disaster event. Hence the study will facilitate understanding not only of the pathogenic factors but also the modifiable conditions which communities can be assisted to address to build resilience and overcome past adversities, thereby averting mental health problems that currently undermine people’s capacity to recover from disaster.

It is recognised that the variables used in this study to select a diverse sample of Victorian communities is limited given the complexity of community experiences. However, the range of variables used in the selection strengthens the likelihood that the participating communities capture a cross section of community issues and outcomes. The study data will also provide an opportunity to examine the range and representativeness of the sample, and the complexity of individual and community experience. It is anticipated that recruitment of study participants, particularly children and young people, will be challenging because of community dislocation, post-trauma sensitivities, research fatigue and the recovery demands on individuals in the fire affected communities. Recruitment may also be difficult in communities which were less affected by the fires because of perceptions of study irrelevance. The community-based participatory approach will however, assist in building trust and rapport and increasing the relevance and appropriateness of the strategies used.

Post-trauma research raises a number of ethical concerns. The instruments chosen for this study are routinely used in general and post-trauma populations. It is well recognised that questioning an individual about their experience of traumatic events may precipitate some emotional reaction. However, the likelihood of being negatively affected by these questions in an ongoing manner is small. The available research evidence suggests that minimal distress of short duration can be expected in a very small minority of participants. Importantly, research has shown that experiencing distress does not affect participants’ willingness to continue in the study [33-35]. Considerable steps have been taken, including development of a suicide risk protocol, to ensure that a range of assistance is available in the unlikely event that participants are distressed by this study.

## Conclusion

This study aims to profile the range of mental health, wellbeing and social responses to the Victorian 2009 bushfires over time. In its use of multiple methodologies and a multidisciplinary/multisectoral team including an extensive network of community partners, it presents a model for future post disaster mixed methods approaches. Affected and unaffected communities will be surveyed to map the predictors and outcomes of mental health and social functioning in children, adolescents, and adults. Conducting the study over five years and focussing on social networks will provide new insights into the interplay between individual and community factors and their influence on recovery from natural disaster over time. The study findings will thereby expand understanding of long term disaster recovery needs for individuals and communities and inform improved policy and practice impacting on health and wellbeing for communities affected by disaster.

## Endnote

^a^This SEIFA index provides a general socio-economic index summarizing a wide range of information on the economic and social resources of the households in an area. The index is based solely on disadvantage and therefore a higher score out of ten (or decile) reflects less disadvantage. A detailed discussion of the mode of collection and analysis of the SEIFA indices can be found at the Australian Bureau of Statistics website (http://www.abs.gov.au/AUSSTATS).

## Competing interests

There are no financial competing interests to report for this study involving the study investigators or study partners. This includes reimbursements, fees, funding, or salary from an organization that may in any way gain or lose financially from the publication of this manuscript, either now or in the future, or financial support for the publication of this manuscript (i.e. the article-processing charge). Nor do any of the investigators have any stocks or shares in an organization that may in any way gain or lose financially from the publication of this manuscript, either now or in the future. Nor do any of the investigators hold or are currently applying for any patents relating to the content of the manuscript, or receiving any reimbursements, fees, funding, or salary from an organization that holds or has applied for patents relating to the content of the manuscript. Consistent with the participatory approach of this study, some of the study authors (JRichardson, VSinnott, GIreton) represent organisations, as listed in their affiliations, that have an interest in or are involved in policy or service delivery that will be informed by the study findings. The remaining authors declare that they have no competing non-financial interests.

## Authors’ contributions

LG was responsible for research project management, drafting the manuscript and community partnership considerations. EW is Principal Investigator of the study and contributed to the cross-disciplinary approach and evidence base contributions. RB contributed to the mental health measures and analysis. PP contributed to the design of the social network analysis component. DL contributed to the development of the social network measures. LH contributed to the development of the community and resilience measures. JR contributed to emergency and service considerations. CMD contributed to child and adolescent considerations. KB contributed to qualitative measures and analysis. ES contributed to recruitment and data collection methods. HG contributed to data management and analysis. VS contributed to policy considerations. GI contributed to disaster recovery issues for communities. DF contributed to post trauma considerations. All of the co-authors contributed to the study design, to the writing of the paper and to the completion of the manuscript. All authors read and approved the final manuscript.

## Pre-publication history

The pre-publication history for this paper can be accessed here:

http://www.biomedcentral.com/1471-2458/13/1036/prepub
